# Standards and Guidelines for Associate's Degree Programs in Gerontology

**DOI:** 10.1093/geroni/igab046.2133

**Published:** 2021-12-17

**Authors:** Suzie Macaluso, Michael Faber

**Affiliations:** 1 Abilene Christian University, Abilene, Texas, United States; 2 Portland Community College, Portland, Oregon, United States

## Abstract

Associate degree programs in gerontology occupy a unique space in higher education. They must prepare students for a wide variety of careers and opportunities from technical and vocational training to preparation for further gerontology education at a four-year college. It is widely known that there is great variability among the numerous associate degree programs in gerontology; this presentation gives an overview of the revised standards and guidelines for associate degree programs. Associate degree programs will vary based on the faculty, the leadership, the program, and institutional support, this presentation discusses best practices for a variety of program types paying particular attention to competency-based educational strategies.

